# Preparation of oral nanoparticles of *Perillae Fructus* oil and prevention application of cold stress in mice

**DOI:** 10.1002/fsn3.3202

**Published:** 2022-12-23

**Authors:** Junfei Xu, Jianxi Zhang, Huiying Lin, Jiayu Zhang, Rong Zhou, Xianjin Wu, Youya Niu, Juzuo Zhang

**Affiliations:** ^1^ College of Biological and Food Engineering Huaihua University Huaihua China; ^2^ Key Laboratory of Research and Utilization of Ethnomedicinal Plant Resources of Hunan Province Huaihua China; ^3^ "Double First‐Class" Applied Characteristic Discipline of Bioengineering in Hunan High Educational Institution Huaihua China; ^4^ School of Basic Medical Sciences Hunan University of Medicine Huaihua China

**Keywords:** cold stress, nanoparticle, *Perillae Fructus* oil, yeast shell

## Abstract

*Perillae Fructus* oil has an important function in relieving cold stress. However, its application in this aspect has still been restricted because of instability and low bioavailability. In this study, *Perillae Fructus* oil was extracted through Soxhlet extraction, analyzed through gas chromatography–mass spectrometry (GC–MS), and nanopackaged into a yeast shell for the preparation of nanoparticles for oral administration. The characteristics of the nanoparticles were investigated using a Malvern zeta‐size nanoinstrument, scanning electron microscopy (SEM), and high‐performance liquid chromatography (HPLC). Then, the roles of orally administered nanoparticles in relieving cold stress were evaluated by investigating blood physiological and biochemical indexes in mice. The results showed that the oil yield from *Perillae Fructus* and shell yield from yeast cells were ~48.37% and ~16.87%, respectively. Approximately 89.21% of the added oil was packaged into the yeast shell to form nanoparticles with an average diameter of 316.74 nm and a surface charge of +2.9 mV. The nanoparticles were stable in simulated gastric acid and could be effectively released in simulated intestinal fluid with an efficiency of ~91.34%. After oral administration of nanoparticles, the mouse blood indexes of white blood cells (WBCs), superoxide dismutase (SOD) activity, and malonaldehyde (MDA) content were recovered compared to those in model mice, with a more remarkable effect than oral administration of free *Perillae Fructus* oil. Overall, the stability and bioavailability were improved by packaging *Perillae Fructus* oil into a yeast shell. These nanoparticles are a new agent for the prevention of cold stress.

## Graphical abstract

The stability and bioavailability of the *Perillae Fructus* oil are improved through nanopackaging into yeast shell, and the oral nanoparticle is a new agent for the prevention of cold stress through strengthening the body capacities of antioxidation and resistance to damage.

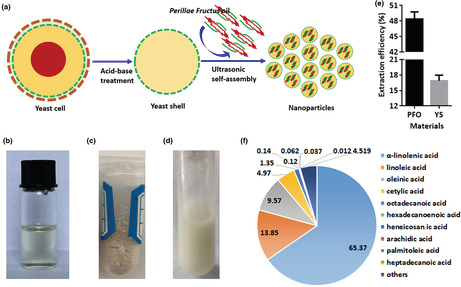

## 1 INTRODUCTION

Cold stress, as a most common element of environmental stress, once overwhelming human thermoregulatory capacity, may lead to the occurrence of the common cold, reduce the body's immunity, make people susceptible to infection, and worsen other chronic diseases (Cheshire Jr., 2016; Vialard & Olivier, 2020). With global warming, the acute drop in temperature phenomenon in unpredictable weather is frequently occurring, and humans have to respond to the increasing occurrence of cold stress that periodically affects human health in clinical practice (Li et al., 2021). Therefore, humans must enhance their ability to cope with cold stress through the improvement of self‐resistance or the intake of preventive agents, especially for infants, older people, and special populations with underlying diseases.

The clinical application of herbal medicine has had many advantages and achievements and has become popular worldwide (Xu et al., 2020). According to the record in “Sheng Nong's Herbal Classic,” the Chinese traditional medicine *Perilla frutescens* (L.) Britt. has an important function in the prevention and treatment of the common cold induced by cold stress, in which *Perillae Fructus* oil plays a key role. Modern medicine studies have also elucidated that *Perillae Fructus* oil can be used to prevent and treat various diseases, such as the common cold, coughing, intestinal disorders, chronic diseases such as diabetes and angiocardiopathy, or mental illnesses such as depression and anxiety, through antioxidant, anti‐inflammatory, and immunoregulatory effects (Ahmed, 2019). *Perillae Fructus* oil has been illustrated to be abundant with omega‐3 (ω‐3) and α‐linolenic acid (ALA) (54–65%), and consumption of *Perillae Fructus* oil is better for reducing many chronic diseases than black sesame supplementation (Koonyosying et al., 2022).

However, there is still a lack of approved products in the medicinal industry, and their clinical application is restricted because of instability, low bioavailability, and inconvenient usage, such as administration via acupuncture points (Yim et al., 2010). The pharmacologically active components in *Perillae Fructus* oil mainly include ω‐3, ALA, flavonoids, sterols, terpenoids, and rosmarinic acid (Ha et al., 2012). These components have common characteristics of volatility, photolysis, oxidizability, and low bioavailability. Therefore, the development of new agents to overcome these defects has become urgent for further medical industrialization.

The biomimetic approach has been demonstrated to be a highly promising method for improving the stability, delivery ratio, and bioavailability of pharmacologically active substances (Heuer et al., 1992). Shells prepared from edible yeast and red blood cells (RBCs) have been illustrated as a highly efficient carrier for delivering drugs to treat tumor and inflammatory diseases through oral administration (Wan et al., 2018; Zhou et al., 2019). In the present study, *Perillae Fructus* oil was separated and encapsulated into a yeast shell to improve its stability and bioavailability, develop a safe and effective new agent, and facilitate the oral administration of cold stress.

## 2 MATERIALS AND METHODS

### 2.1 The extraction of *Perillae Fructus* oil through the Soxhlet extraction method

The samples of mature *Perillae Fructus* were purchased from herb markets in Huaihua city, and its dried powder was prepared by crushing with a universal pulverizer and sifting out with a 60‐mesh screen. The powders (100 g) and anhydrous ether (300 ml) were successively loaded into the cellulose thimbles of Soxhlet extraction equipment. The extraction was performed with standard circulating water at 40°C for 5 h. The filtrates were concentrated and dried to constant weight for recycling ether and dehydrating. The oil yield was analyzed after three repetitions.

### 2.2 Analysis of *Perillae Fructus* oil through GC–MS

The analysis of *Perillae Fructus* oil in fatty acid composition was conducted through gas chromatography–mass spectrometry (GC–MS) in a DB‐WAX capillary column (30 m × 0.25 mm × 0.25 μm) with helium at a flow rate of 1.0 ml/min using a temperature program of heating from 50°C for the first 1 min until the temperature reached 240°C for the final 5 min at a rate of 5°C/min. Analysis was conducted for 40 min in split mode (80:1). The injector temperature was 280°C, and the ion source temperature was 230°C. The analysis was performed with an electron impact (EI) source ionization energy of 70 eV in single‐ion monitoring (SIM) mode. The instrumental conditions were a quadrupole temperature of 150°C, an auxiliary heating temperature of 250°C, a transmission line temperature of 280°C, and a mass scanning range of 35–455 *m/z*. Qualitative analysis was conducted in the reference range of ion proportions of ±20%.

### 2.3 Culture of yeast cells and separation of yeast shells

The yeast cells were recovered from the lyophilized yeast powder using sterile yeast peptone dextrose (YEPD) medium, including 20 g of tryptone, 10 g of yeast extract powder, and 20 g of glucose in 1 L of deionized water. A single colony of yeast was cultured and selected using YEPD solid medium dishes for further expanded culture. The yeast cells at 20 g were collected from the cultured solution and washed with 20 ml of deionized water 1 time by centrifugation at 3438 *g* for 10 min. The yeast cells, resuspended using 1 ml of deionized water, were added to 200 ml of 1 M NaOH and incubated at 80°C for 1 h under stirring, followed by the same conditional washing three times. The collected samples were again dispersed in 200 ml of HCl (pH 3.0) and incubated at 55°C for 1 h under light stirring, followed by the same conditional washing three times. The collected samples were washed with 10 ml of isopropyl alcohol four times, followed by rinsing with 10 ml of acetone twice. Then, the collected samples, yeast shell, were dried through vacuum lyophilization and used in the following experiments.

### 2.4 Nanoparticle preparation of *Perillae Fructus* oil with yeast shell

The yeast shell was dissolved in normal saline at a 4 mg/ml concentration. The solution of yeast shell was preprocessed under ultrasonic conditions with 90 W power at 20 kHz for 2 min (running 5 s, interval 5 s), followed by addition into *Perillae Fructus* oil (10 mg/ml) at a final concentration of 2 mg/ml, and processing under the same ultrasonic conditions for 10 min. The solution was kept quiescent in the dark at room temperature for 16 h of self‐assembly. The prepared nanoparticles were stored at room temperature for the following experiments.

### 2.5 Characteristics analysis of nanoparticles

The prepared solution was added to an electrode cup of a Malvern zeta‐size nanoinstrument for the detection of the size and surface charge of the nanoparticles. The surface charge and size of the nanoparticles were also detected through SEM. The concentration of free *Perillae Fructus* oil in the supernatant of the prepared solution was detected through HPLC after centrifugation to determine the loading ratio. The antioxidation capacity of the nanoparticles and free *Perillae Fructus* oil was determined through the DPPH method.

### 2.6 Oral application of nanoparticles in mice under cold stress

Animal experiments were approved by the Animal Ethical and Welfare Committee of Huaihua University (Huaihua: 2021(H031755)). Twelve male and 12 female KM mice were purchased from the Center of Experimental Animals at the Hunan University of Medicine and housed in a clean room with free access to food and water on a 12 h light/12 h dark cycle. All mice were randomly assigned to four groups through the number lottery method, including the normal control (NC) group, cold stress model (CSD) group, treatment with *Perillae Fructus* oil (TPFO) group, and treatment with nanoparticles (TNPs) group. There were six mice in each group, including three male mice and three female mice, and mice of different sexes were separated and housed in different cages. All mice were weighed every day. The mice in the NC and CSD groups were administered an equal volume of normal saline. The mice in the TPFO and TNP groups were supplied with free *Perillae Fructus* oil or nanoparticles of *Perillae Fructus* oil at a dose of 250 mg/kg body weight each day (mg/(kg·d)), respectively. The supplement was administered intragastrically for 9 successive days. In the last 3 successive days (days 7–9), the mice in the NC group were kept in a normal environment at 26°C, and the mice in the CSD, TPFO, and TNP groups were placed in a 4°C chamber for 6 h (from 8 am to 2 pm) each day for acute cold exposure. Body temperature was detected at 2‐h intervals in this experimental process. At the end of the experimental day (day 9), blood samples were collected for content determination of components and cells.

### 2.7 Detection of blood index

The composition of blood cells, including RBCs, white blood cells (WBCs), and platelets (PLTs), was determined through an automatic blood cell analyzer. The activity of superoxide dismutase (SOD) and the content of malondialdehyde (MDA) in blood were investigated using an ELISA kit (Cat. No.: 69‐30,017, and Cat. No.: 69‐21,068, MSKBIO Co. Ltd., Wuhan, China) according to the manufacturer's instructions. The fasting blood glucose levels were monitored through a Sano blood glucose meter with test strips (Cat. No.: WP‐PE‐VQW, Sanocare Co. Ltd.).

### 2.8 Statistical analysis

Statistical analysis was performed by one‐way ANOVA and two‐tailed Student's *t*‐test after normal distribution tests using the Statistical Package for Social Science (SPSS, Version 19.0; SPSS Inc, Chicago, IL, USA). The final data are expressed as the mean ± standard deviation (SD) with statistical significance at *p* < .05 or *p* < .01.

## 3 RESULTS

### 3.1 The preparation of the nanoparticle solution

**FIGURE 1:**
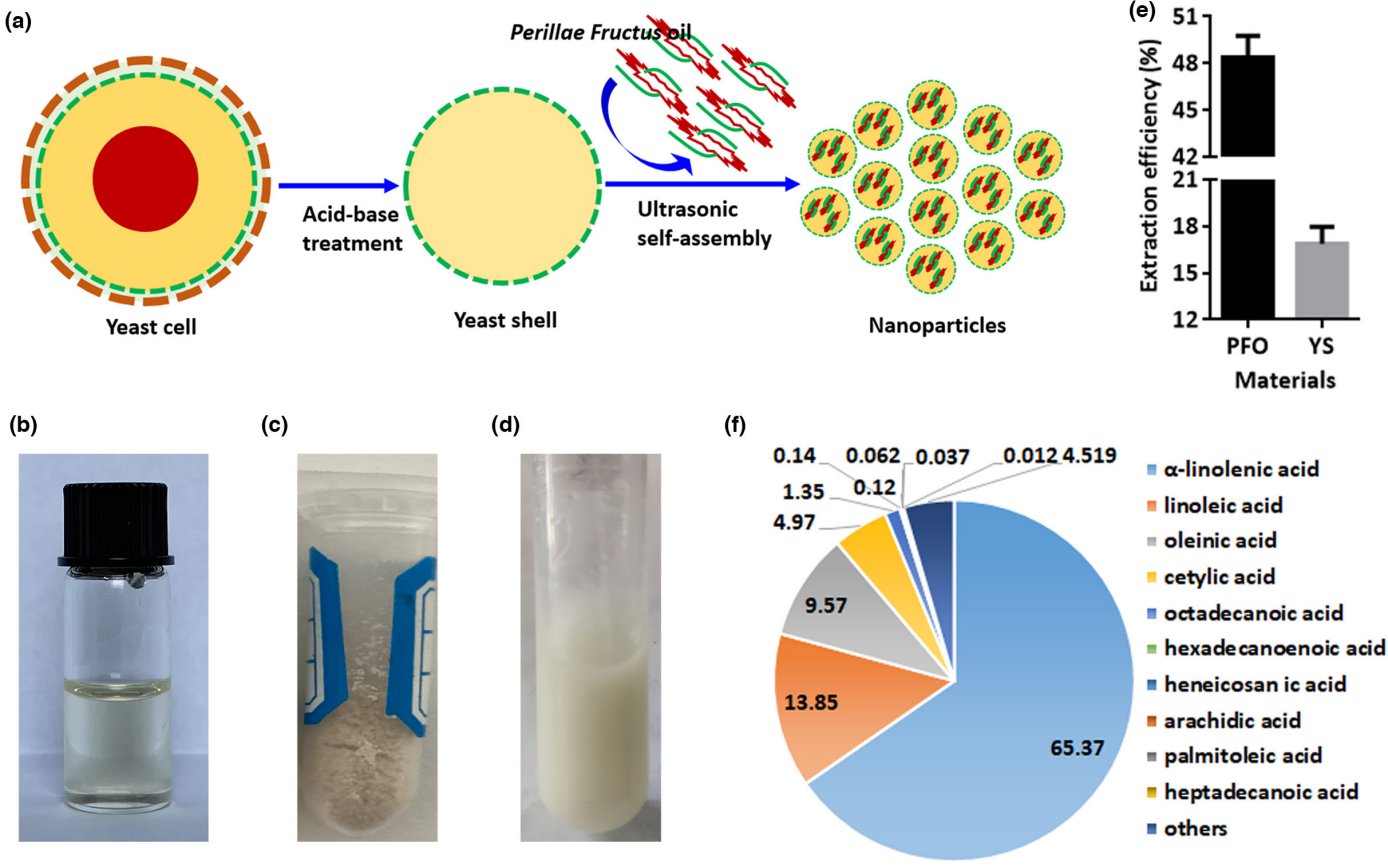
Preparation of *Perillae Fructus* oil nanoparticles. (a) The preparation schematic of *Perillae Fructose* oil nanoparticles. (b) The oil was extracted from *Perillae Fructus* at a ratio of ~48.37%. (c) The yeast shell was separated from yeast cells with a ratio of ~16.87%. (d) A solution of nanoparticles in which *Perillae Fructus* oil was nanopackaged into yeast shells. (e) The extracted ratio of *Perillae Fructus* oil and yeast shells. (f) The fatty acid composition of *Perillae Fructus* oil, in which the unsaturated fatty acid content was over 89.08%.

The nanoparticles of *Perillae Fructus* oil were prepared according to the schematic in Figure 1a. First, the oil was extracted from *Perillae Fructus* at a ratio of 48.37% (Figure 1b, e), and the yeast shell was separated from yeast cells at a ratio of 16.87% (Figure 1c, e). Then, the nanoparticle solution was prepared with a milk‐white appearance through ultrasonic self‐assembly (Figure 1d). Moreover, the GC–MS results showed that the *Perillae Fructus* oil was composed of 95.48% fatty acids and 4.52% others. There were mainly 10 kinds of fatty acids in the *Perillae Fructus* oil, including 65.37% α‐linolenic acid, 13.85% linoleic acid, 9.57% oleinic acid, 4.97% cetylic acid, 1.35% octadecanoic acid, 0.14% hexadecanoic acid, 0.12% heneicosanoic acid, 0.062% arachidic acid, 0.037% palmitoleic acid, and 0.012% heptadecanoic acid (Figure 1f).

### 3.2 Nanoparticle characteristics

**FIGURE 2:**
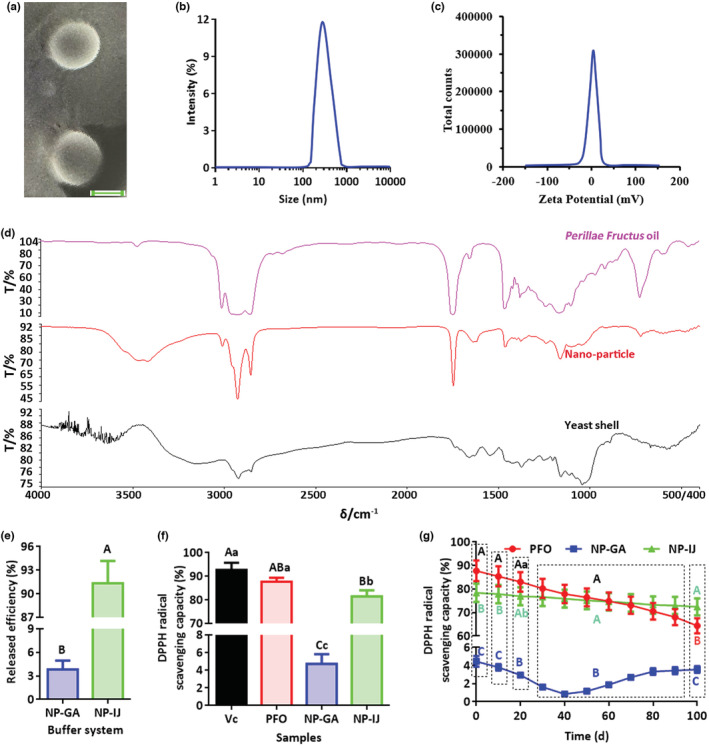
Characteristic analysis of *Perillae Fructus* oil nanoparticles. (a) The SEM appearance of *Perillae* Fructose oil nanoparticles (scale bar: 200 nm). (b) The size distribution of nanoparticles with an average diameter of 316.74 nm. (c) The zeta potential of nanoparticles with a surface charge of +2.9 mV. (d) The absorption spectra of *Perillae Fructus* oil, nanoparticles, and yeast shell. (e) The release efficiency of nanoparticles in gastric acid (3.83%) or intestinal fluid (~91.34%). (f) Analysis of the antioxidation activity of *Perillae Fructus* oil (PFO), nanoparticles in gastric acid (NP‐GA), and nanoparticles in intestinal fluid (NP‐IJ) through the DPPH method, in which vitamin C (Vc) was used as an indicator. (g) Analysis of the preservation stability of *Perillae Fructus* oil (PFO), nanoparticles in gastric acid (NP‐GA), and nanoparticles in intestinal fluid (NP‐IJ) through the DPPH method. The different capital letters indicate extremely significant differences (*p* < .01). The different lowercase letters indicate significant differences (*p* < .05). Vc, vitamin C; PFO, *Perillae Fructus* oil; NP‐GA, nanoparticle in gastric acid; NP‐IJ, nanoparticle in intestinal fluid.

After ultrasonic self‐assembly, the nanoparticles were relatively homogeneous (Figure 2a) with an average diameter of 316.74 nm (Figure 2b) and a surface charge of +2.9 mV (Figure 2c). According to SEM, the *Perillae Fructus* oil was successfully nanopackaged into the yeast shell (Figure 2d) with an efficiency of approximately 89.21% to form nanoparticles. The nanoparticles were stable in a simulated condition of gastric acid and could be effectively released in a simulated condition of intestinal fluid with an efficiency of ~91.34% (Figure 2e). Compared with vitamin C, free *Perillae Fructus* oil had a similar DPPH radical scavenging capacity, significantly higher than that of nanoparticles in intestinal fluid buffer and significantly higher than that of nanoparticles in gastric acid buffer (Figure 2f). Under storage conditions for 100 days, the antioxidation of *Perillae Fructus* oil in nanoparticles was more stable than that in a free state, some of which was significantly oxidized (Figure 2g). These results illustrated that the *Perillae Fructus* oil could be nanopackaged into the yeast shell for the preparation of nanoparticles, which would contribute to oil stability.

### 3.3 Oral nanoparticles relieve cold stress in mice

**FIGURE 3:**
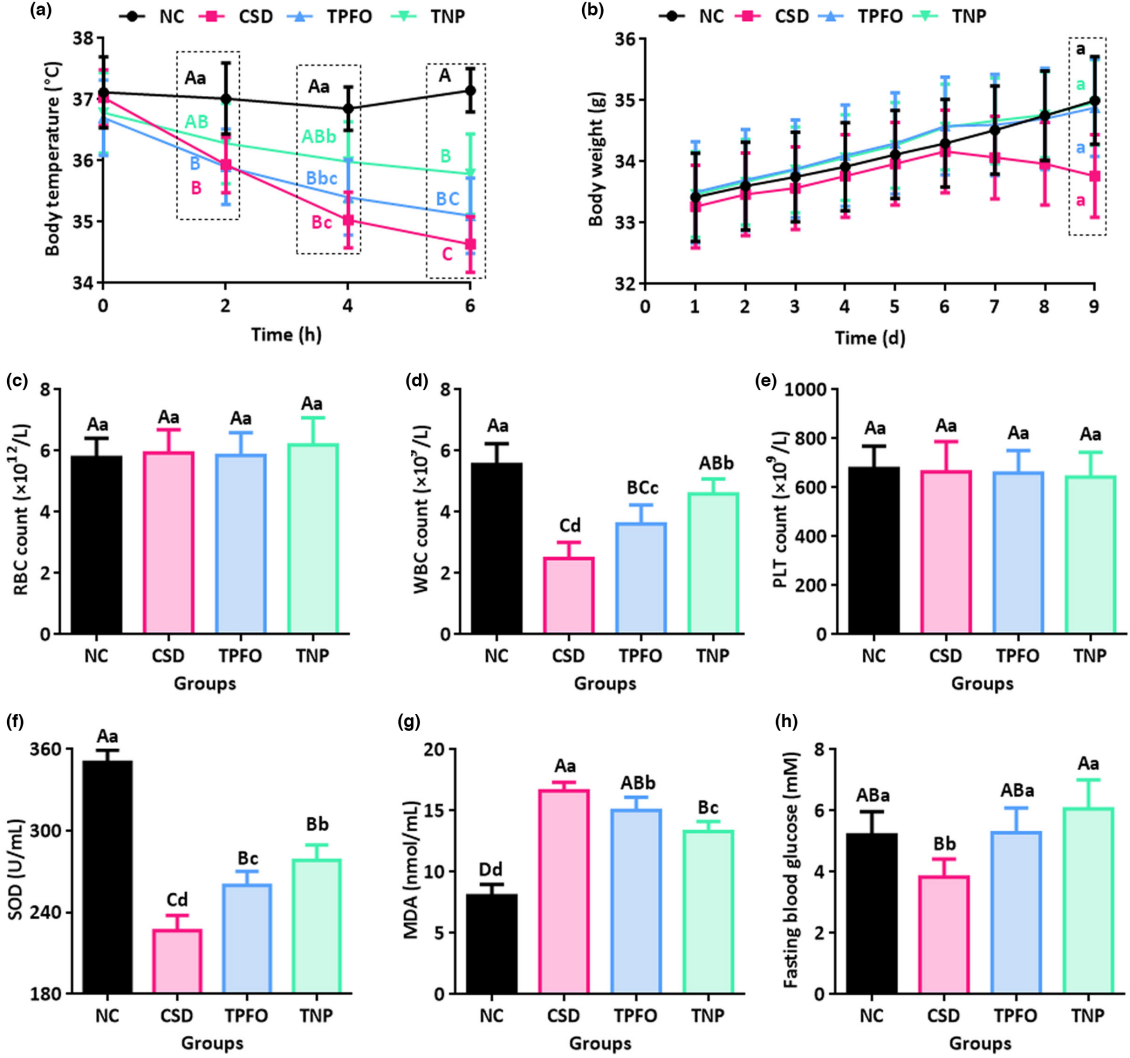
Oral nanoparticles relieve cold stress in mice. (a) Mouse body temperature under cold stress conditions in the NC, CSD, TPFO, and TNP groups. (b) Analysis of body weight during the experimental process. (c) Blood index analysis of red blood cells on the deadline day. (d) Blood index analysis of white blood cells on the deadline day. (e) Blood index analysis of platelets on the deadline day. (f) Analysis of SOD levels in the blood. (g) Analysis of MDA levels in the blood. (h) The fasting blood glucose level. The different capital letters indicate extremely significant differences (*P* < .01). The different lowercase letters indicate significant differences (*p* < .05). NC, negative control; TPFO, treatment using *Perillae Fructus* oil; TNP, treatment using nanoparticles; SEM, scanning electron microscopy; WBC, white blood cell; SOD, superoxide dismutase; MDA, malonaldehyde.

Under cold stress conditions, the mouse body temperature in the TPFO and TNP groups was closer to normal temperature (NC group) than that in the CSD group, and the mice in the TNP group had stronger resistance to cold stress, suggesting that dietary supplementation with *Perillae Fructus* oil was beneficial for thermostasis and that the new nanoparticle agent had a better effect than free *Perillae Fructus* oil (Figure 3a). After cold stress, the mouse body weight in the CSD group was slightly decreased, and the increasing trend in mouse body weight in the TPFO and TNP groups was inhibited. On the last day, the mouse body weight in the NC, TPFO, and TNP groups was significantly higher than that in the CSD group (Figure 3b). There was no significant difference in the RBC and PLT blood indexes among the four groups (Figure 3c, e). The mouse WBC count and serum SOD activity in the CSD, TPFO, and TNP groups were significantly decreased compared with those in the NC group, and they were significantly recovered when mice were administered free *Perillae Fructus* oil or nanoparticles, which had a more notable effect (Figure 3d, f). The mouse serum MDA level in the CSD, TPFO, and TNP groups was significantly increased compared with that in the NC group, and it was significantly recovered when the mice were administered free *Perillae Fructus* oil or nanoparticles, which had a more notable effect (Figure 3g). The mouse fasting blood glucose level in the CSD group was significantly decreased compared with that in the NC group, and it was significantly recovered when the mice were administered free *Perillae Fructus* oil in the TPFO group and maintained a higher level when the mice were administered nanoparticles in the TNP group (Figure 3f). These results demonstrated that the nanoparticles had a better role in the prevention of cold stress than free *Perillae Fructus* oil, and suggested that it could improve the biocompatibility to combine the *Perillae Fructus* oil with the yeast shell.

## 4 DISCUSSION

In the unsaturated fatty acid family, both ω‐6 and ω‐3 fatty acids are essential for human health. ω‐6 fatty acids are more easily obtained from a normal diet because it is more abundant in daily cooking oil than ω‐3 fatty acids (Asif, 2011; Goulet et al., 2010). However, a high ratio of ω6:ω3 may lead to or exacerbate the occurrence of chronic diseases, and current studies have shown that dietary supplementation with ω‐3 has benefits in preventing and controlling chronic diseases, such as tumorigenesis, diabetes, and cognitive impairments (Wong et al., 2016; Yam et al., 2019). In this study, we found that the unsaturated fatty acid content was over 89.08%, and the saturated fatty acid content was low at 6.45%. Interestingly, α‐linolenic acid in *Perillae Fructus* oil, as a kind of ω‐3, was over 65.37%. Therefore, *Perillae Fructus* oil is a very promising material in the dietary and medical application of ω‐3 supplements.

Although *Perillae Fructus* oil has potential application value in diet and medicine, there are still many practical production dilemmas. On the one hand, α‐linolenic acid is easily destroyed in the processes of extraction and preservation. In this study, the antioxidation capacity of free *Perillae Fructus* oil was significantly decreased. However, the nanoparticles could efficiently reserve the antioxidation capacity of *Perillae Fructus* oil. Therefore, encased *Perillae Fructus* oil with a yeast shell is more stable than free *Perillae Fructus* oil, and the nanoparticle is a new promising agent. On the other hand, α‐linolenic acid is easily destroyed by gastric acid and is difficult to absorb and utilize in the free state. In this study, to overcome these defects, *Perillae Fructus* oil was linked with the yeast shell, nanopackaged into the yeast shell, and blocked its interaction with oxygen. The good biocompatibility of the yeast shell was used to improve the bioavailability of *Perillae Fructus* oil. Therefore, the nanoparticles showed a better role in the prevention and treatment of common cold and cold stress.

Several studies have demonstrated that the defense system, such as interleukin level, SOD activity, and MDA content, is influenced during the cold stress process (Guo et al., 2022; Lee et al., 2014; Zhou et al., 2006). In the present study, the blood SOD activity, as a defense indicator against cold stress both in humans and animals (Zhao et al., 2014), was improved in mice after administration of free *Perillae Fructus* oil or the prepared nanoparticles. Moreover, the blood content of MDA, as a damage indicator, was reduced in mice orally administered free *Perillae Fructus* oil or the prepared nanoparticles. Therefore, dietary supplementation with *Perillae Fructus* oil, especially when formulated in nanoparticles, may improve the body's capacities of antioxidation and resistance to damage.

In conclusion, the stability and bioavailability of *Perillae Fructus* oil are improved by nanopackaging into yeast shells, and orally administered nanoparticles are a new agent for the prevention of cold stress.

## CONFLICT OF INTEREST

The authors declare that they have no conflicts of interest.

## ETHICAL APPROVAL

Animal experiments were approved by the Animal Ethical and Welfare Committee of Huaihua University (Huaihua: 2021(H031755)).

## Data Availability

The data that support the findings of this study are available on request from the corresponding authors.
